# Supplementary material for: Mapping evidence on factors contributing to maternal and child mortality in sub-Saharan Africa: A scoping review protocol
Source: PLoS One. 2022 Aug 10;17(8):e0272335. doi: 10.1371/journal.pone.0272335 (PMC9365121; doi:10.1371/journal.pone.0272335)
Supplement: S1 File — (PDF) [file pone.0272335.s002.pdf]

UNIVERSITY OF KWAZULU-NATAL
INYUVESI YAKWAZULU-NATALI

Public Library of Science
1160 Battery St, Suite 225
San Francisco, CA 94111

26 January 2021

Dear Sir/Madam

Request to waive article - processing charges (Journal: PLOS ONE)

The University of KwaZulu-Natal Research Office supports the cost of publication fees for all Department Of Higher Education and Training (DHET) accredited journal articles. As per University policy, the maximum threshold for journals ranked under Quartile One as per the Scimago institutions ranking database is ZAR 15,000.00, such as the Journal: PLOS One; the researcher must cover all excess costs. If the journal is removed from the DHET listing the researcher will be responsible for all costs incurred.

We request that PLOS waive the article processing charges over and above the ZAR 15,000.00 limit offered by the University policy.

Yours Sincerely,

Prof Urmilla Bob (PhD)
University Dean of Research and Full Professor in Geography
University of KwaZulu-Natal
Tel: +27 31 260 3270
Fax: +27 31 260 3325
Email: bobu@ukzn.ac.za
