# Supplementary material for: Mapping evidence on factors contributing to maternal and child mortality in sub-Saharan Africa: A scoping review protocol
Source: PLoS One. 2022 Aug 10;17(8):e0272335. doi: 10.1371/journal.pone.0272335 (PMC9365121; doi:10.1371/journal.pone.0272335)
Supplement: S2 File — (DOCX) [file pone.0272335.s003.docx]

**S2 file: Pilot search in PubMed database**

| **Keyword search** | **Date of search** | **Search engine** | **Number of publications retrieved** |
| --- | --- | --- | --- |
|  | 2021/04/15 | PubMed | 1120 |
| ((("maternal mortality"[MeSH Terms] OR "mothers"[MeSH Terms] OR "mothers"[All Fields] OR "maternal"[All Fields]) AND ("mother child"[Journal] OR ("mother"[All Fields] AND "child"[All Fields]) OR "mother child"[All Fields]) AND ("infant mortality"[MeSH Terms] OR ("infant"[All Fields] AND "mortality"[All Fields]) OR "infant mortality"[All Fields]) AND ("factor"[All Fields] OR "factor s"[All Fields] OR "factors"[All Fields]) AND ("analysis"[MeSH Subheading] OR "analysis"[All Fields] OR "determination"[All Fields] OR "determinant"[All Fields] OR "determinants"[All Fields] OR "determinate"[All Fields] OR "determinated"[All Fields] OR "determinates"[All Fields] OR "determinating"[All Fields] OR "determinations"[All Fields] OR "determine"[All Fields] OR "determined"[All Fields] OR "determines"[All Fields] OR "determining"[All Fields])) OR "causes of death"[All Fields]) AND ("africa south of the sahara"[MeSH Terms] OR ("africa"[All Fields] AND "south"[All Fields] AND "sahara"[All Fields]) OR "africa south of the sahara"[All Fields] OR ("sub"[All Fields] AND "saharan"[All Fields] AND "africa"[All Fields]) OR "sub-saharan africa"[All Fields]) AND 1990/01/01:2021/04/15[Date - Publication] |  |  |  |
