# Supplementary material for: Mapping evidence on factors contributing to maternal and child mortality in sub-Saharan Africa: A scoping review protocol
Source: PLoS One. 2022 Aug 10;17(8):e0272335. doi: 10.1371/journal.pone.0272335 (PMC9365121; doi:10.1371/journal.pone.0272335)
Supplement: S3 File — (DOCX) [file pone.0272335.s004.docx]

| S3 file: Data charting form |  |  |  |
| --- | --- | --- | --- |
| Author and date  Title  Country  Aims  Study population  Sample size  Study design  Study setting  Key findings |  |  |  |
|  |  |  |  |
